# Differential effects of trait-like emotion regulation use and situational emotion regulation ability across the affective and anxiety disorders spectrum: a transdiagnostic examination

**DOI:** 10.1038/s41598-024-76425-7

**Published:** 2024-11-04

**Authors:** Dirk Adolph, Jürgen Margraf

**Affiliations:** Department of Psychology, Mental Health Research and Treatment Center, Ruhr-University Bochum, Massenbergstraße 9-13, 44787 Bochum, Germany

**Keywords:** Emotion regulation, Cognitive behavior therapy, Anxiety, Depression, Psychopathology, Transdiagnostic approach, Prediction of treatment outcome, Human behaviour, Comorbidities

## Abstract

Here, we investigated the association of different emotion regulation (ER) indices with symptom severity across a large transdiagnostic sample of patients with emotional disorders (*cross-sectional approach*) and the predictive validity these ER indices have for the outcome of routine care CBT (*longitudinal approach*). We assessed the trait-like use of adaptive (reappraisal) and maladaptive (suppression, externalizing behaviors) ER strategies via questionnaire as well as the situational ability to regulate emotions with an experimental ER paradigm. Psychopathology was assessed dimensionally using the depression, anxiety, and stress scale. C*ross-sectionally* symptom severity was predicted by less trait-like use of adaptive and more trait-like use of maladaptive ER strategies, but no associations were found for situational ER ability. This association was more pronounced for depression and stress symptoms rather than anxiety symptoms. In a striking dissociation, the *longitudinal analyses* revealed the reverse picture: Better situational ER ability, but not trait-like use of ER strategies was associated with less symptom severity after the CBT treatment. Our data argues in favor of a distinction between trait-like and situational ER abilities in individuals with emotional disorders, highlighting challenges in applying adaptive ER strategies in daily life despite demonstrating intact ER skills in experimental settings. Our findings also inform transdiagnostic models of psychopathology and suggest that distress/depression rather than anxiety symptomatology to be driving forces for the occurrence of ER deficits across the depression/anxiety disorders spectrum.

## Introduction

Although diagnostic manuals (like DSM-V and ICD 11) list anxiety and affective disorders as distinct disorder categories, they show exceptionally high comorbidity rates^1–3^ and significant symptom-overlap^4^. To consolidate the wealth of empirical research on symptom overlap between anxiety and affective disorders so far, theoretical models^5^ consistently associate both syndromes with a broad range of affective dysregulations, like enhanced susceptibility towards experiencing negative affect (observable across the full range of affective and anxiety disorders), threat responses or hyperarousal (associated with anxiety disorders including panic disorder or phobias) and low positive affect (most prominently associated with depression, generalized anxiety or social phobias). One promising candidate to disentangle the mechanisms underlying these common and distinct emotional symptoms across the affective and anxiety disorders spectrum is emotion regulation (ER). ER refers to the extrinsic and intrinsic processes responsible for monitoring, evaluating, and modifying emotions according to a person’s goals^6^. ER can thereby influence which emotion is experienced, how intense, and how long it is experienced and how it is expressed^7^. Problems in the identification of ER goals, or the selection of appropriate ER strategies and their implementation can be observed across a variety of mental disorders^8^. For example, a growing number of meta-analyses consistently demonstrate that in comparison to healthy controls less frequent use of adaptive ER strategies (e.g. acceptance, problem solving, reappraisal) and more frequent use of maladaptive strategies (e.g., avoidance, rumination, suppression), is evident in most psychopathologies (substance use^9,10^; eating disorders^9,11^; psychosis^12^, borderline personality disorder^13^, bipolar disorder^14^, PTSD^15^), including depression and anxiety^9,16–20^. Consequently, several authors have defined ER as a transdiagnostic process relevant for the development and/ or maintenance^8,21–23^, as well as for the treatment^24,25^ across mental disorders.

A transdiagnostic approach, as suggested in recent transdiagnostic models of psychopathology like the Research Domain Criteria (RDoC)^26^ or the Hierarchical Taxonomy of Psychopathology (HiTOP)^27^, is especially promising to disentangle the intertwined mechanisms underlying the extensive symptom comorbidity and emotional dysregulations across the affective and anxiety disorders spectrum. However, current studies often do not consider the transdiagnostic significance of ER. Thus, a direct comparison of the extend of ER problems across the affective and anxiety disorders has only rarely been realized. For example, many studies on ER still include a single diagnostic group and a control group only (i.e. either patients with depressive or anxiety disorder). Since the primary goal of research on transdiagnostic processes is to identify which dysfunctional processes cut across diagnostic categories (i.e., are transdiagnostic) and which do not (i.e., are disorder specific), research on the transdiagnostic significance of ER need to include more than one disorder category. Moreover, there is still a lack of studies analyzing clinical symptoms of affective and anxiety disorders dimensionally. Indeed, such an approach is needed to quantify the dose-dependent relationship between symptom severity and ER (e.g., do ER deficits become more severe as the symptom-severity increases?)^21^. In addition, the dimensional assessment of symptoms is necessary to better understand functional overlap between affective and anxiety disorders at the symptom level, an approach crucial to elucidate the mechanistic underpinnings of excessive symptom comorbidity observed within the affective and anxiety disorders spectrum.

In fact, we were able to identify only five studies so far who assessed ER using a transdiagnostic approach according to the above-mentioned criteria (inclusion of more than one diagnostic group, analysis of symptoms dimensionally) across the affective and anxiety disorders, and these are methodologically too heterogeneous to draw reliable conclusions. In brief, three of these five studies assessed the trait-like use of ER strategies and symptoms of depression and anxiety via questionnaire in healthy participants yielding contradictory results. One found that^28^ the trait-like use of cognitive ER was associated with symptoms of depression *and* anxiety. However, this study did not differentiate between adaptive and maladaptive ER strategies. A second study assessed the association between internalizing symptomatology and ER and found that higher fear was associated with an enhanced choice of maladaptive ER strategies in an ER paradigm, while distress was associated with an enhanced choice of reappraisal^29^. The third study assessed an adolescent sample and found that less trait like use of reappraisal was associated with depression symptomatology. No associations were found for anxiety symptoms^30^. None of these three studies included patient samples, largely limiting their clinical significance. To the best of our knowledge only two studies to date assessed ER simultaneously in a patient’s sample diagnosed for depression and anxiety^31^. The first study found that patients regardless of the diagnose, reported less frequent use of adaptive ER strategies as compared to a healthy control group. No differences were found between depression and anxiety. This study neither assessed dysfunctional ER strategies nor included dimensional measures of depression and anxiety symptomatology. Finally, the second study^32^ found that patients with anxiety disorders and depression both showed dysfunctional activation in ER related brain areas during a reappraisal-based ER task. In that study, results were mainly driven by anxiety rather than depression symptomatology.

### The current study

In sum, converging evidence suggest that affective and anxiety disorders are associated with a range of ER difficulties^9,19,20,33^. However, as outlined above, studies so far do not allow for a differential understanding of the interrelationship between ER-dysfunction and symptomatology within the affective and anxiety disorders spectrum. However, this is important. Comorbidity within the anxiety and depression spectrum is present on the syndrome level and (perhaps even more common) on the symptom level. That is, in clinical praxis, overlapping symptom patterns in patients from the depression/anxiety spectrum are the rule rather than the exception. For example, a patient with a diagnosis of panic disorder may additionally appear to suffer from several symptoms of depression no matter whether these are falling below or beyond the threshold for a full-blown depressive disorder, or vice versa. Most past research into ER deficits in depression and anxiety has compared patients from one disorder category with a sample of healthy controls leaving it largely unclear, if ER deficits are more related to depression symptomatology, anxiety symptomatology or equally related to both symptom complexes. Given the high comorbidy of depression and anxiety, specificity of ER deficits can be analyzed with a transdiagnostic approach only, assessing the symptom clusters of interest in a dimensional manner within a sample of participants showing a large range of the symptoms of interest. This allows for the calculation of specific relationships between symptom clusters and ER strategies while controlling for concurrent symptoms and other ER strategies, and thus allows for the identification of ER deficits which a specific for certain symptom clusters or essentially transdiagnostic.

In the current study, we assessed (1) the frequency of use of adaptive and maladaptive ER strategies (i.e. trait-like use of ER strategies) and (2) the ability to implement these strategies to effectively regulate emotions (i.e. situational ER ability) across a large transdiagnostic sample of participants showing a wide range of anxiety and depressive symptomatology.

We assessed the trait-like frequency of ER strategy use (adaptive and maladaptive) via questionnaire. This was accomplished with the Negative Affect Repair Questionnaire^34^. It consists of a reappraisal and suppression scale comparable to the ER scales assessed with the Emotion Regulation Questionnaire (ERQ)^35^, as well as a scale assessing a set of externalizing ER strategies covering maladaptive and dysfunctional response-focused emotion regulation strategies like for example substance use, aggressive or self-harming behavior, which are associated with negative psychological health outcomes^37^ and stress^38–40^.

We assessed the situational ability to effectively achieve ER with an emotion regulation paradigm using threatening and sadness inducing film clips and assessed objective (facial Electromyography) as well as subjective emotion responses. Participants were diagnosed with a standardized semi-structured clinical interview and symptoms of anxiety and affective disorders were assessed dimensionally using the Depression, Anxiety and Stress Scale^41^ (See Online Supplemental Materials for detailed information on the conceptual fit of the DASS with transdiagnostic models of depression and anxiety). With this *cross-sectional* approach, we tested the transdiagnostic association of state and trait indices of ER with the symptom-clusters across the emotional disorders’ spectrum.

Additionally, we also assessed the predictive validity ER has for the outcome of routine-care Cognitive Behavior Therapy (CBT) as provided in our outpatient center. CBT is among the most efficious treatments for emotional disorders^42^ and the most widespread standard treatment at German outpatient centers^43^. In fact, CBT augmented with an explicit ER training has been proven effective in decreasing emotion disorder symptomatology, including negative affect^44^, anxiety and stress^45^. However, most standard CBT manuals do not explicitly include direct ER skills trainings^33,46^. Indeed, while one overarching goal of CBT for emotional disorders is to reduce negative affect, this is typically accomplished via a range of therapeutic interventions like exposure and cognitive restructuring but without directly accomplishing ER skills training. Thus, mostly CBT target ER processes rather indirectly^33^. So far, only very few studies have assessed ER as a causal factor for the outcome of routine care CBT for anxiety and depression^33^, and to the best of our knowledge no study so far directly compared the predictive validity of ER for *routine care* CBT-outcome across a transdiagnostic sample of patients from the anxiety and affective disorders spectrum. However, this is important, since longitudinal studies only allow for the assessment of ER as a causal factor for the prospective development of psychopathology^33^. Therefore, to enhance our understanding of the significance ER has for routine care CBT, after completing ER assessment all patients underwent CBT as routinely carried out at our center. With this *longitudinal* approach, we thus probed the significance of state and trait indices of ER as a causal factor in *routine care* CBT across the emotional disorders’ spectrum.

Based on previous meta-analytical data showing ER deficits in nearly all psychological disorders, we await cross sectional associations of ER indices with the entire range of emotion disorder symptoms. In specific, based on previous research we hypothesize that more severe depression and anxiety symptomatology is transdiagnostically associated with less frequent use of reappraisal and more frequent use of suppression. We further await more frequent use of externalizing strategies to be transdiagnostically associated with elevated levels of stress symptomatology. Moreover, based on previous research with healthy participants showing more pronounced associations of ER difficulties with depression/ distress symptomatology we hypothesize stronger associations of ER indices with depression rather than anxiety symptomatology across the transdiagnostic sample. Because only very few studies to date assessed ER as a causal factor, no strong hypotheses could be drawn concerning the longitudinal approach. However, because of previous findings showing that CBT enhanced with emotion regulation skills training reduces emotion disorders symptomatology, we await predictive validity of ER indices for depression, anxiety and stress symptomatology.

## Results

### Experimentally assessed ER—proof of principle

**Fig. 1:**
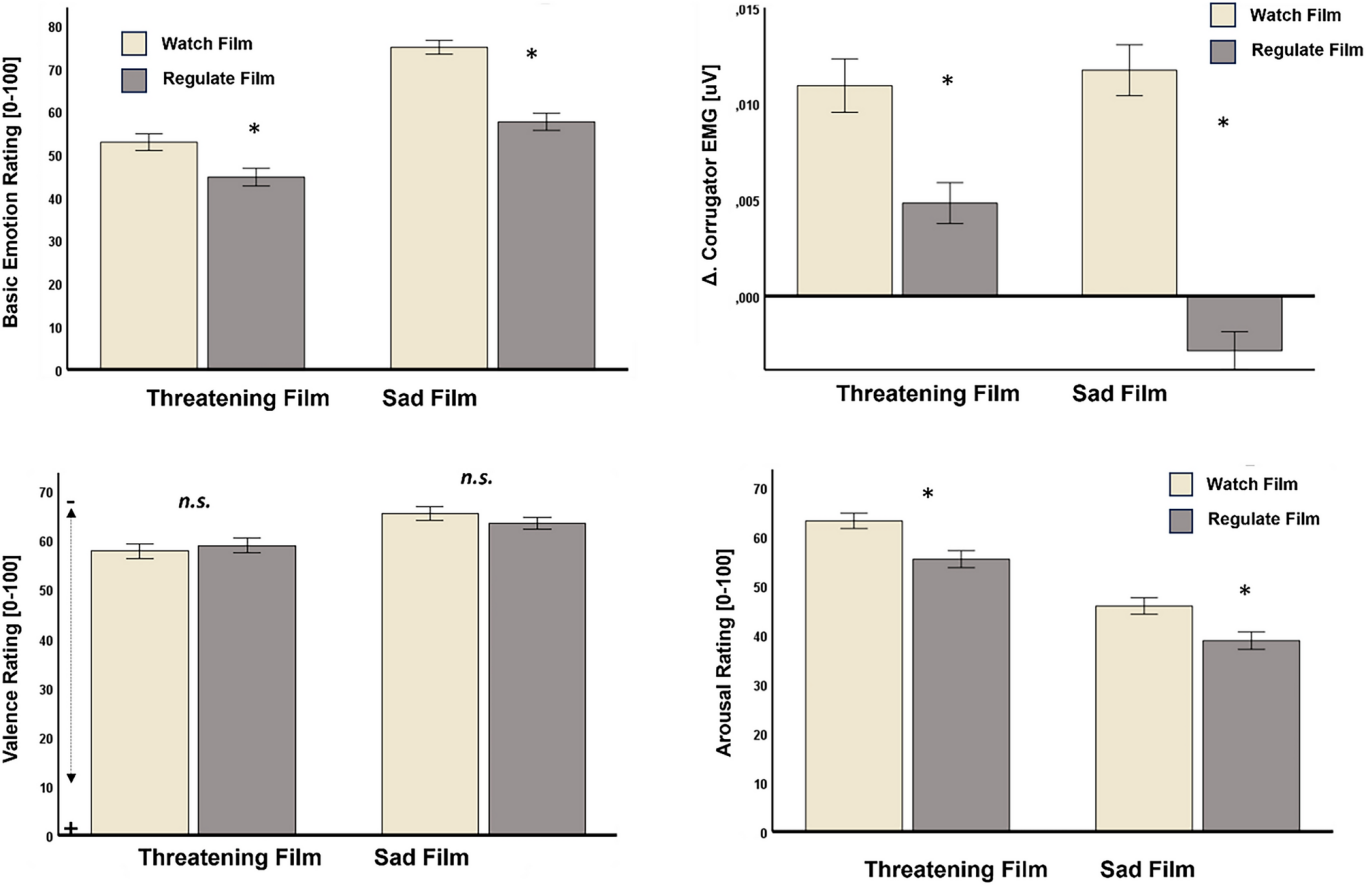
Results indicate successful experimentally assessed ER: Participants reported less intense basic emotions (upper left panel) and showed less intense activity of the M. corrugator supercilii in the emotion regulation condition (upper right panel), as well as less arousal (lower right panel) as compared to the passive viewing condition towards both, the threatening, and the sad film clip. However, there were no differences in valence ratings between the passive viewing and the emotion regulation condition.

Concerning emotion intensity ratings, participants rated the threatening film clip as significantly less anxiety-inducing during the emotion regulation than during the passive viewing condition, F(1,247) = 16.33, p < 0.001, η^2^ = 0.062, n = 248. Likewise, participants rated the sad film clip as significantly less sadness-inducing during the emotion regulation condition, than during the passive viewing condition, F(1,247) = 87.27, p < 0.001, η^2^ = 0.261, n = 248 (see Fig. 1 upper left panel). In line with this, participants felt less aroused towards both film clips during the emotion regulation condition, as compared to the passive viewing condition (threatening film clip: F[1,247] = 20.59, p < 0.001, η^2^ = 0.077, n = 248, sad film clip: F[1,247] = 16.90, p < 0.001, η^2^ = 0.064, n = 248, see Figure 1 lower right panel), and displayed significantly less M. corrugator supercilii activity towards the threatening and sad film clips during the emotion regulation, as compared to the passive viewing condition (Threatening film clip: F[1,189]33.48, p < 0.001, η^2^ = 0.150, n = 195; sad film clip: F[1,189] = 120.00, p < 0.001, η^2^ = 0.388, n = 195). (See Fig. 1 upper right panel). There were no significant differences in subjective ratings of valence between the passive viewing and the emotion regulation condition for both film clips (threatening film clip: F[1,247] = 0.56, p = 0.457, n = 248; sad film clip: F[1,247] = 2.12, p = 0.147, n = 248).

### Cross sectional analyses: transdiagnostic prediction of symptom severity

Table 1 shows bivariate correlations between ER indices and symptoms of depression, stress and anxiety across the entire transdiagnostic sample. Results indicate that less trait-like use of reappraisal and more trait-like use of externalizing ER strategies were associated with higher depression, anxiety and stress symptomatology. More use of suppression was associated with higher depression and stress symptomatology. No associations were found between symptom severity and the situational ER ability.

**Table 1:** Transdiagnostic correlations between depression, anxiety and stress symptoms and indices of emotion regulation.

	DASS_Anxiety_	DASS_Stress_	NARQ_Reapraisal_	NARQ_Suppression_	NARQ_Externalizing_	ER_threat_	ER_sad_
DASS_Depression_	0.594** (n = 250)	0.638** (n = 250)	0.495** (n = 222)	0.273** (n = 222)	0.438** (n = 222)	0.113 (n = 246)	− 0.124 (n = 246)
DASS_Anxiety_	–	0.537** (n = 250)	− 0.220** (n = 222)	0.110 (n = 222)	0.259** (n = 222)	− 0.054 (n = 246)	− 0.088 (n = 246)
DASS_Stress_		–	− 0.380** (n = 222)	0.166* (n = 222)	0.549** (n = 222)	− 0.003 (n = 246)	− 0.061 (n = 246)
NARQ_Reapraisal_			–	− 0.351** (n = 222)	− 0.385** (n = 222)	0.137* (n = 246)	0.077* (n = 246)
NARQ_Suppression_				–	0.201* (n = 222)	− 0.127 (n = 246)	0.101 (n = 246)
NARQ_Externalizing_					–	− 0.049 (n = 246)	− 0.076( n = 246)
ER_threat_						–	0.187** (n = 246)

**p < 0.01; *p < 0.05.

#### Associations of ER indices with depression symptomatology

Table 2 gives an overview about the regression model predicting depression symptomatology across the transdiagnostic sample. In brief, anxiety and stress symptoms alone explained about 51.3% of variance in depression symptomatology across participants (Regression model 1). ER indices explained additional 8.3% of variance in depression symptomatology (Regression model 2). In sum, more severe depression symptoms were associated with less trait use of reappraisal, and more use of suppression strategies. Situational ability to regulate threatening and sad emotions (ER_sad_, ER_threat_) did not significantly predict pre-treatment levels of depression in the transdiagnostic sample.

**Table 2:** Regression analysis predicting depression symptom-severity with emotion regulation indices and levels of anxiety and depression.

Effect	*B*	*t*	*p*	95% CI		*VIF*
				*LL*	*UL*	
Model 1: F(2,218) = 113.55, p < 0.001, R^2^ = 0.513, n = 219						
Intercept	0.291	0.49	0.625	− 0.879	1.461	–
DASS_Anxiety_	0.506	6.36	< 0.001**	0.349	0.663	1.40
DASS_Stress_	0.513	8.17	< 0.001**	0.389	0.636	1.40
Model 2: F(7,218) = 44.40, p < 0.001, ΔR^2^ = 0.083, n = 219						
Intercept	3.856	2.34	0.020	0.602	7.109	–
DASS_Anxiety_	0.491	6.67	< 0.001**	0.346	0.636	1.41
DASS_Stress_	0.376	5.54	< 0.001**	0.242	0.510	1.9
ER_threat_	− 0.461	− 1.27	0.204	− 1.174	0.252	1.05
ER_sad_	− 0.486	− 1.20	0.231	− 1.284	0.311	1.04
NARQ_Reappraisal_	− 0.394	− 4.29	< 0.001**	− 0.575	− 0.213	1.392
NARQ_Suppression_	0.158	2.00	0.046*	0.003	0.313	1.18
NARQ_Externalizing_	0.080	0.92	0.357	− 0.091	0.251	1.55

**p < 0.01; *p < 0.05.

#### Associations of ER indices with anxiety symptomatology

Table 3 gives an overview about the regression model predicting anxiety symptomatology. Depression and stress symptoms alone explained 39.1% of variance in anxiety symptomatology (Regression model 1). However, neither trait use of ER strategies (reappraisal, suppression, externalizing behavior), nor situational ER ability (ER_sad_, ER_threat_) significantly predicted pre-treatment anxiety symptomatology (Regression model 2).

**Table 3:** Regression analysis predicting anxiety symptom-severity with emotion regulation indices and levels of depression and stress.

Effect	*B*	*t*	*p*	95% CI		*VIF*
				*LL*	*UL*	
Model 1: F(2,218) = 71.04, p < 0.001, R^2^ = 0.391, n = 219						
Intercept	0.565	1.218	0.225	− 0.350	1.480	–
DASS_Depression_	0.194	3.541	< 0.001**	0.086	0.302	1.73
DASS_Stress_	0.312	6.361	< 0.001**	0.215	0.408	1.73
Model 2: F(7,218) = 21.17, p < 0.001, ΔR^2^ = 0.016, n = 219						
Intercept	− 0.381	− 0.27	0.789	− 3.181	2.420	
DASS_Depression_	0.227	3.80	< 0.001**	0.109	0.345	2.06
DASS_Stress_	0.355	6.67	< 0.001**	0.250	0.460	2.04
ER_threat_	0.027	0.09	0.929	− 0.581	0.636	1.06
ER_sad_	0.030	0.09	0.930	− 0.650	0.710	1.05
NARQ_Reappraisal_	0.109	1.35	0.178	− 0.050	0.269	1.50
NARQ_Suppression_	− 0.045	− 0.66	0.509	− 0.178	0.088	1.20
NARQ_Externalizing_	− 0.088	− 1.20	0.233	− 0.233	0.057	1.55

**p < 0.01; *p < 0.05.

#### Associations of ER indices with stress symptomatology

Table 4 gives an overview about the regression models predicting stress symptomatology. A total of 44.8% of variance in the DASS stress subscale was explained by depression and anxiety symptomatology (Regression model 1). Regression model 2 revealed, that ER indices explained additional 9.3% of variance in stress symptomatology. In brief, more frequent trait-like use of externalizing behaviors predicted higher levels of stress symptomatology across the transdiagnostic sample. The remaining ER indices did not significantly predict pre-treatment stress symptomatology (ER_sad_, ER_threat,_ trait use of reappraisal or suppression).

**Table 4:** Regression analysis predicting stress symptom-severity with emotion regulation indices and pre-treatment levels of depression and anxiety.

Effect	*B*	*t*	*p*	95% CI		*VIF*
				*LL*	*UL*	
Model 1: F(2,218) = 88.43, p < 0.001, R^2^ = 0.448, n = 219						
Intercept	4.774	10.39	< 0.001	3.868	5.680	–
DASS_Depression_	0.461	8.17	< 0.001**	0.350	0.572	1.57
DASS_Anxiety_	0.283	3.54	< 0.001**	0.125	0.441	1.57
Model 2: F(7,218) = 21.17, p < 0.001, ΔR^2^ = 0.093, n = 219						
Intercept	4.594	2.960	0.003	1.534	7.655	
DASS_Depression_	0.338	5.54	< 0.001**	0.218	0.458	2.16
DASS_Anxiety_	0.282	3.80	< 0.001**	0.136	0.429	1.59
ER_threat_	0.530	1.55	0.123	− 0.145	1.204	1.04
ER_sad_	0.180	0.47	0.641	− 0.578	0.938	1.05
NARQ_Reappraisal_	− 0.047	− 0.52	0.605	− 0.226	0.132	1.51
NARQ_Suppression_	− 0.045	− 0.59	0.555	− 0.193	0.104	1.20
NARQ_Externalizing_	0.460	6.06	< 0.001**	0.310	0.610	1.33

**p < 0.01; *p < 0.05.

### Longitudinal analyses: transdiagnostic prediction of treatment outcome

#### Prediction of post-treatment anxiety

Pretreatment levels of anxiety symptomatology significantly predicted post-treatment anxiety and explain a total of 31.7% of variance in post-treatment anxiety. ER indices explained additional 8.6% variance in post-treatment anxiety (Table 5). In detail, less post-treatment anxiety symptomatology was predicted by better ER ability to regulate threatening emotions (i.e. ER_threat_) and less frequent use of externalizing strategies (Table 5). Neither situational ability to regulate sad emotions, nor frequency of reappraisal or suppression use significantly predicted post CBT levels of anxiety.

**Table 5:** Regression predicting post treatment symptom-severity of depression with emotion regulation indices and pre-treatment depression.

Effect	*B*	*t*	*p*	95% CI		*VIF*
				*LL*	*UL*	
Model 1: F(1,117) = 75.78, p < 0.001, R^2^ = 0.395, n = 118						
Intercept	1.440	1.81	0.074	− 0.140	3.020	–
DASS_Depression_	0.635	8.71	< 0.001**	0.490	0.779	1.00
Model 2: F(1,117) = 14.17, p < 0.001, ΔR^2^ = 0.039, n = 118						
Intercept	1.525	0.60	0.547	− 3.479	6.528	–
DASS_Depression_	0.588	7.47	< 0.001**	0.432	0.744	1.19
ER_threat_	− 1.141	− 1.92	0.058^#^	− 2.321	0.039	1.04
ER_sad_	0.326	0.55	0.584	− 0.849	1.501	1.02
NARQ_Reappraisal_	− 0.090	− 0.62	0.536	− 0.377	0.197	1.26
NARQ_Suppression_	0.037	0.29	0.776	− 0.218	0.291	1.13
NARQ_Externalizing_	0.163	1.37	0.172	− 0.072	0.397	1.19

**p < 0.01; *p < 0.05; #p<.100

#### Prediction of post-treatment stress

Pre-treatment stress symptoms explained 39.4% of variance in post-treatment stress. ER indices explained additional 3.7% of variance. Thereby, less severe post-treatment stress symptomatology was predicted by better situational ability to regulate threatening emotions (i.e. ER_threat_). Neither trait use of any ER strategy, nor the situational ability to regulate sad emotions significantly predicted post treatment stress (Table 6).

**Table 6:** Regression predicting post-treatment symptom-severity of anxiety with emotion regulation indices and pre-treatment anxiety.

Effect	*B*	*t*	*p*	95% CI		*VIF*
				*LL*	*UL*	
Model 1: F(1,117) = 53.75, p < 0.001, R^2^ = 0.317, n = 118						
Intercept	1.096	2.024	0.045	0.023	2.168	–
DASS_Anxiety_	0.561	7.331	< 0.001**	0.409	0.712	1.00
Model 2: F(1,117) = 12.50, p < 0.001, ΔR^2^ = 0.086, n = 118						
Intercept	1.692	0.942	0.348	− 1.868	5.252	–
DASS_Anxiety_	0.544	7.310	< 0.001**	0.396	0.691	0.97
ER_threat_	− 1.003	− 2.319	0.022*	− 1.860	− 0.146	0.96
ER_sad_	− 0.753	− 1.751	0.083	− 1.605	0.099	0.98
NARQ_Reappraisal_	− 0.020	− 0.195	0.845	− 0.224	0.184	0.83
NARQ_Suppression_	− 0.097	− 1.051	0.296	− 0.280	0.086	0.90
NARQ_Externalizing_	0.181	2.138	0.035*	0.013	0.348	0.87

**p < 0.01; *p < 0.05.

#### Prediction of post-treatment depression

Regression model 1 revealed that pre-treatment levels of depression symptomatology explained 39.5% of variance in post-treatment depression. ER indices explained additional 3.9% of variance. Regression model 2 revealed that at trend level (*p* = 0.058) less severe post-treatment depression symptomatology was predicted by better situational ability to regulate threatening emotions (i.e. ER_threat_). None of the other ER indices significantly predicted post treatment depression (Table 7).

**Table 7:** Regression predicting post treatment symptom-severity of stress with emotion regulation indices and pre-treatment stress.

Effect	*B*	*t*	*p*	*95% CI*		*VIF*
				*LL*	*UL*	
Model 1: F(1,117) = 75.42, p < 0.001, R^2^ = 0.394, n = 118						
Intercept	2.047	2.31	0.022	0.294	3.800	–
DASS_Stress_	0.643	8.68	< 0.001**	0.496	0.789	1.00
Model 2: F(1,117) = 13.99, p < 0.001, ΔR^2^ = 0.037, n = 118						
Intercept	4.229	1.84	0.068	− 0.318	8.776	–
DASS_Stress_	0.638	7.64	< 0.001**	0.472	0.803	1.30
ER_threat_	− 1.267	− 2.40	0.018*	− 2.314	− 0.220	1.06
ER_sad_	− 0.001	− 0.01	0.998	− 1.031	1.029	1.02
NARQ_Reappraisal_	− 0.110	− 0.88	0.379	− 0.357	0.137	1.21
NARQ_Suppression_	− 0.111	− 0.99	0.327	− 0.333	0.112	1.13
NARQ_Externalizing_	0.037	0.33	0.740	− 0.184	0.259	1.38

**p < 0.01; *p < 0.05.

## Discussion

The current study investigated how different emotion regulation (ER) indices relate to symptom severity in individuals with emotional disorders using both cross-sectional and longitudinal approaches. Cross-sectionally, higher symptom severity was transdiagnostically associated with less trait-like use of adaptive ER strategies and more trait-like use of maladaptive ER strategies, but situational ER abilities did not show significant associations. Longitudinally, better situational ER ability predicted reduced symptom severity post-CBT, highlighting the importance of specific ER skills in therapeutic outcomes.

### Cross sectional associations of ER indices

As expected, across the transdiagnostic sample, bivariate correlations showed significant associations between more severe depression, anxiety, and stress symptoms with less frequent use of adaptive (i.e., reappraisal) and more frequent use of maladaptive (suppression and externalizing behaviors) ER strategies. At first glance, these findings are in line with the multitude of research showing ER difficulties in most disorders from the emotion disorders spectrum, including affective and anxiety disorders^9,16–20^. However, emotional disorders are highly comorbid on both the syndrome, as well as the symptom level^1–4^. To accommodate to these prerequisites, and to identify the unique variance the different emotion disorders’ symptoms have in common with ER indices, we calculated additional regression analyses controlling for co-occurring symptoms. These analyses painted a more differential picture and revealed that depression and stress symptoms explained most of the variance anxiety symptoms had in common with trait-like use of ER strategies. That is, after controlling for depression and stress, the association between anxiety symptoms and ER strategy use was no longer significant. Rather, more severe depression symptoms were uniquely associated with less frequent use of reappraisal and more frequent use of suppression. Thus, our data point to symptoms of depression rather than anxiety as driving forces for the manifestation of ER difficulties across typical emotion disorders. These findings are in line with current data on healthy populations showing that less trait like use of reappraisal is associated with depression symptomatology rather than anxiety symptomatology^30^ in an adolescent sample, as well as with a study showing stronger associations between maladaptive trait like use of ER strategies and depression as compared to anxiety symptomatology^28^ in adults. In sum, our data thus extend these findings and show that this depression-specific association can be extended to clinical populations.

Importantly, more robust depression-specific associations were found previously for other indexes of self-regulation. For example, we previously reported a more robust association between depression (as compared to anxiety) symptoms with vagally mediated heart rate variability (vmHRV) in a treatment seeking sample of patients with emotion disorders^47^. vmHRV indexes the activity of a top-down regulation system^48,49^ involved in the organisms flexible physiological responding to emotional or stress-related environmental demands, and is associated with behavioral flexibility, cognitive executive functioning and self-regulation^50–52^. Moreover, recently Granros and Co-workers^53^ found that across a large transdiagnostic patients’ sample, less flexible affective reactivity (in terms of blunted late positive event related potentials, LPP, towards emotional pictures) was associated with a pooled distress/misery factor including measures of depression, suicidality, or lassitude, but not with a pooled fear/anxiety factor^53^. Thus, converging evidence from previous work and the current study suggest symptom-specificity of deficits in emotion self-regulation and cognitive control. Moreover, these cognitive control deficits likely constitute a transdiagnostic psychopathological mechanism, which is more strongly associated with depression symptomatology rather than anxiety symptomatology across the emotion disorders spectrum.

Interestingly, we found that cross-sectionally, more severe stress symptomatology was associated with an enhanced use of externalizing ER strategies. The externalizing behaviors scale assesses a range of maladaptive actions like self-harming, aggressive behavior and substance use, practices that have been previously identified as maladaptive stress regulation strategies^54,55^ and are negatively associated with the frequency of using adaptive ER strategies^10,56^. Negative correlations between the use of externalizing strategies and reappraisal have also been found in the current study, indicating that the use of these maladaptive behaviors is accompanied with limited access to adaptive ER strategies^56,57^. This suggest that across the emotional disorders spectrum maladaptive behavioral ER strategies might serve as a substitutional ER strategy to reduce stress-related symptomatology in the absence of the ability to recruit adaptive ER strategies.

Taken together, the current findings extend recent meta-analytical evidence showing that trait-like use of ER strategies is a common phenomenon across the spectrum of emotional disorders including depression *and* anxiety disorders and suggest that the differences between patients and healthy controls found in previous studies might not be manifestations of the disorders per se but are rather tied to the pattern of emotional symptoms present within the individual patients. Thus, our data shows, that emotion regulation disabilities are not direct consequences of distinct disorders or disorder subgroups but occur in a symptom-specific manner across the emotion disorders spectrum. Noteworthy, the current data add also to our understanding of mechanisms underlying the internalizing disorders spectrum in light of current transdiagnostic models of emotional disorders, like the Hierarchical Taxonomy of Psychopathology^27,58^. These models argue towards a dimensional structure of psychopathology to explain comorbidity and symptom-overlap between disorders. Within these empirically derived models, distress and fear constitute distinct sub-spectra of an underlying internalizing spectrum consisting of disorders accompanied with emotional dysfunctions. Within the scope of these models, our data strongly suggest emotion regulation as an underlying transdiagnostic mechanism explaining co-occurring emotion symptoms. Moreover, our data indicate that the driving force underlying these ER deficits across the internalizing disorders spectrum are symptoms from the distress/depression sub-spectrum, rather than the fear sub-spectrum. Clearly, our sample included patients with anxiety and depressive disorders, covering only a sub-spectrum of the internalizing dimension, and future research is needed to extend these findings to the entire spectrum. However, nonetheless, the current findings help to understand the underlying mechanisms of comorbidity between depression and anxiety disorders, and the temporal dynamics of anxiety and depressive symptomatology, i.e. why some depressive disorders are more likely to precede the onset of anxiety while others onset secondary to anxiety^59^.

Contrary to the trait-like use of ER strategies our cross-sectional analysis did not reveal any significant associations between the actual ER ability in response to emotion inducing film clips and the severity of depression anxiety or stress symptoms. This finding is in line with previous research reporting no significant differences between patients and healthy controls in their actual ability to regulate emotions^60–63^ and a recent meta-analysis showing no brain abnormalities during reappraisal both at the transdiagnostic level or for specific disorder categories^64^. Taken together, this reveals a remarkable dissociation between the situational ability to successfully implement adaptive ER strategies to down-regulate emotions and the trait-like failure to successfully recruit these potential skills. Thus our, and previous data highlight the importance of situational factors and context variables in the assessment of ER difficulties across psychopathology. The assessment of ER in everyday life using ecological momentary assessments could be beneficial in the attempt to enhance ecological validity in the attempt to clarify the association of ER deficits and psychopathology^33^.

### Prediction of treatment outcome

Contrary to our cross-sectional analysis, the longitudinal approach shows that the situational ability to reappraise negative emotions ,but not the trait like use of ER strategies, predicts the outcome of CBT. Notably, this association was present for the full range symptoms, showing that in our transdiagnostic sample better ability to reappraise negative emotions induced by emotional film clips predicted lower post treatment levels of anxiety, stress, *and* depression. These data correspond to previous research showing that adaptive emotion regulation abilities are broadly related to psychological well-being^65–67^ and positive indicators of mental health, life satisfaction and positive affect^68,69^. Furthermore, our data extend findings from longitudinal studies indicating predictive validity of emotion regulation for the course of anxiety and depression symptoms over time^70–72^, as well as research demonstrating the predictive validity of the situational ER ability for the outcome of an anxiety treatment^73^. Taken together, the current data suggest the situational ability to effectively recruit emotion regulation as one underlying mechanism for the treatment of emotional disorders^33^. Indeed, supporting this notion, studies have found that specific ER skills training improves the outcome of a psychotherapeutic treatment^74^. In the same vein, emotion regulation centered treatments like the “Unified Treatment for Emotional Disorders”^75^ have been proven effective in treating emotional disorders like depression and anxiety. In specific, a recent meta-analysis found that the unified protocol effectively reduces depression *and* anxiety symptomatology across the entire emotion disorders spectrum^76^. Interestingly, while specialized treatments like the unified protocol incorporate specific modules directly targeting ER skills, typical CBT protocols like those used in the present study, often do not directly implement emotion regulation skills trainings^33^. Rather, these protocols mostly target emotional dysregulation indirectly via exposure training or restructuring and other cognitive techniques. Interestingly, cognitive restructuring, fear extinction and emotion regulation (especially when incorporating reappraisal as the primary ER strategy) are overlapping constructs sharing relevant features, for example, largely overlapping neural networks centering on prefrontal brain structures^77–80^. Furthermore, they incorporate mechanisms of cognitive control^81^ and target emotional dysfunctions by inhibiting unwanted thoughts or emotional reactions^78,82–84^. Thus, in sum, our data highlight the pivotal role the ability to actively engage these cognitive control processes has for the outcome of cognitive behavior therapy^25,84–86^.

As compared to the predictive validity of situation ER ability, associations between trait-like use of ER strategies and treatment outcome were rather sparse. Indeed, we did not find any associations between the outcome of CBT with the trait-like use of reappraisal (for a comparable finding see^87^). Rather, our data indicate that more frequent use of externalizing strategies is associated with less reduction of anxiety symptoms after CBT, indicating that beyond its cross-sectional association with stress symptomatology, enhanced use of maladaptive behaviors like self-harming or substance abuse is also disadvantageous for the outcome of CBT. The current findings are in line with previous studies showing that the trait-like use of maladaptive ER strategies is associated with less favorable treatment outcome in anxiety and affective disorders^72,88^ and with research showing that a change in maladaptive ER strategies predict better treatment outcome for anxiety disorders^89^ and further highlights the maladaptive nature of the trait-like use of externalizing behaviors.

### Limitations

First, several patients (~ 29%) did not attend the post-treatment questionnaire assessment or terminated the treatment prematurely, resulting in a smaller power of our longitudinal analyses. However, the current response-rate to post-treatment data assessment is comparable to previous studies in outpatient settings (^90^), and patients who dropped out did not differ from those finishing the study in terms of gender, age, or their depression, anxiety or stress level. Moreover, due to our large initial patient sample, the final *N* for the longitudinal analysis was still considerably high with considerable statistical power. Thus, it is unlikely that the current effects were significantly affected by the drop-outs. Related, we did not assess a control group. Thus, although previous studies have shown the effectiveness of routine care CBT in our and other German outpatient centers^90,91^, we cannot entirely rule out the possibility, that the simple passage of time could be responsible for symptom improvement. Second, we did not assess income, education and socioeconomic status, putatively limiting generalizability of the current results. Third, intercorrelations between the DASS subscales were high, ranging from 0.537 to 0.638, and thus specificity of the three scales in terms of assessing different symptom clusters might be limited. However, these intercorrelations are comparable to those found previously in studies using the DASS in clinical samples^40,92^ and similar to those obtained with psychometrically comparable instruments in clinical research and practice, for example the Beck Depression Inventory and the Beck Anxiety Inventory^41,93^. Moreover, our analyses showed an acceptable amount of 50%, 60% and 55% unique variance unexplained by co-occurring symptomatology for the depression, anxiety and stress scales, respectively. Additionally, results obtained in the current study, were largely in line with theoretical considerations and previous research suggesting reliable results. However, future research should consider using additional self-report instruments covering the core symptoms of the anxiety and depression disorders spectrum while showing better discrimination between scales. For example, the Mood and Anxiety Symptoms Questionnaire^94^ has been used in another study on transdiagnostic associations of ER deficits^28^ and showed intercorrelations of around 0.30 between its anhedonic depression and anxious arousal subscales. Fourth, we did not assess the activity of the sympathetic nervous system, nor asked participants to document the exact regulation strategy they adopted during the emotion regulation task. Therefore, we cannot entirely rule out that at least some participants engaged in ER strategies other than reappraisal (e.g. Suppression). However, one of our previous studies using comparable emotion regulation instructions assessed the participants’ strategies used to down-regulate at the end of the experiment. These data indicated compliance with instructions of the vast majority of participants^95^. Moreover, in the current study participants clearly reported feeling significantly less aroused during the emotion regulation condition, as compared to the passive viewing condition. Ratings of arousal towards emotional stimuli have repeatedly been shown to covary with measures of sympathetic arousal (i.e. the skin conductance response) (for a prototypical study see^96^), suggesting subjective ratings of arousal as a rough indicator of sympathetic responding. In addition, participants reported experiencing the target emotion significantly less intensely during the regulation as compared to the passive viewing condition (ratings of fearfulness and sadness). However, although in sum this suggest successful downregulation beyond simple suppression in the current study, future research should include measures of sympathetic activation and should ask participants to report on ER strategies used upon the end of the study. Fifth, due to the multimethod approach in the current study, we decided to limit the assessment of ER strategies. That is, we cannot rule out that if we had used a larger range of ER strategies (for example including experiential avoidance or rumination) anxiety symptoms would have shown larger associations with respect to the trait-like use of ER strategies.

## Conclusion

In sum, the current study has important implications concerning the relationship between emotion disorders and emotion regulation. The present data argues in favor of a distinction between the general trait-like ER ability and the situational ER ability (i.e. the potential for successfully achieving the individual ER goals in a certain situation ^97^. That is, despite intact situational ability to use adaptive ER strategies in the laboratory, patients show deficits in implementing these strategies in their daily life and more frequently rely on non-adaptive ER strategies to manage negative affect. Given the significance of situational ability to implement adaptive ER strategies like reappraisal for the outcome of therapeutic interventions, future research is needed to identify the context factors that keep patients from achieving successful ER in everyday life. Such information has the potential to I) further improve treatment approaches based on ER strategies like the unified treatment for the spectrum of emotional disorders^98^ and to II) advance the current attempts to develop individually tailored psychotherapeutical treatments^99^.

## Materials and methods

### Sample characteristics

To assure clinical significance (in terms of clinically significant depressive and anxiety symptomatology) and a considerable range of symptom severity, we recruited a mixed transdiagnostic sample of patients with diagnoses of anxiety or depressive disorders along with healthy participants. A total of N = 223 patients attending for treatment at the Mental Health Research and Treatment Center at Bochum University participated. Diagnoses were obtained by trained and certified psychotherapists using a standardized semi-structured interview for DSM-IV disorders (Diagnostisches Interview für Psychische Störungen, DIPS)^100^. The diagnoses obtained with the DIPS show very good interrater-reliability (Kappa between κ = 0.72 and κ = 0.92;^101,102^ and its validity has been previously verified^103^. Of the N = 223 patients, n = 27 did not qualify for a diagnosis of a depressive or an anxiety disorder and had to be excluded from the study. Within the final sample, a total of n = 103 patients had a depressive disorder and n = 93 patients an anxiety disorder. Of these, a total of *n* = 22 patients with depression had a comorbid anxiety disorder and a total of *n* = 31 patients with an anxiety disorder had a comorbid depressive disorder. A total of n = 89 patients took psychotropic medication. In addition to the patient sample, a total of *n* = 60 healthy adults (HC) participated in the current study. Diagnoses of healthy controls were obtained with a brief semi-structured interview for DSM-IV disorders^104^ and via self-report. No healthy control participant had to be excluded due to a current or history of mental disorders. The final sample consisted of n = 95 male and n = 179 female participants, with an age ranging from 18 to 73 years (M = 36.2, SD = 13.26). All participants were Caucasian and recruited from the Ruhr Area in Germany. All participants gave written and informed consent to procedures. The study was conducted in accord with the Declaration of Helsinki and was approved by the local ethics committee of the Faculty of Psychology at Ruhr-University Bochum (Approval Number: Votum046). Comprehensive sample descriptions can be found in Table 8 and Table S1.

**Table 8:** Descriptive statistics of depression, anxiety and stress symptomatology of the current sample.

	*M*	*SD*	Range
DASS_Depression_ *M (SD)*	7.81	6.12	0–21
DASS_Anxiety_ *M (SD)*	5.04	4.36	0–21
DASS_Stress_ *M (SD)*	9.78	5.38	0–21

*DASS* depression anxiety and stress scale.

### Questionnaires

#### Depression anxiety and stress scale

Depression and anxiety symptomatology, as well as pre-post symptom change after CBT was assessed with the Depression, Anxiety and Stress Scale^41^. We used the DASS, because it has been developed to assess the full range of core symptoms of anxiety and depression while providing maximum discrimination between scales^40^. Moreover, although not entirely equivalent, the three DASS subscales assess the three symptom domains described in the tripartite model of depression and anxiety (i.e. anhedonic depression, anxiety/ arousal, negative affect/distress)^40^. Thus, using the DASS maximizes discrimination of symptom clusters as well as conformity with empirically derived theoretical models of depression and anxiety. The validity of the DASS-21 for clinical populations^40,92^ and to assess treatment outcome^105,106^ has been demonstrated. The DASS has 21 items with 4-point Likert-scales (0 = did not apply to me at all to 3 = applied to me most of the time). The three subscales reached excellent internal consistency (in terms of Cronbach α) in the current sample (stress α = 0.890, depression α = 0.935, anxiety α = 0.837).

#### Negative affect repair questionnaire

For the questionnaire-based emotion regulation assessment, the Negative Affect Repair Questionnaire was used^36^. The NARQ consists of 17 items assessing the frequency of use of the three emotion regulation strategies Reappraisal, Suppression, and Externalizing Strategies on 5 point scales (0 = never–4 = always). The scales Reappraisal and Suppression are comparable to the ER scales assessed with the Emotion Regulation Questionnaire^34^. The Externalizing Strategies scale consist of a number of items assessing maladaptive and dysfunctional response-focused emotion regulation strategies^35^ like for example substance use, aggressive or self-harming behavior, which are associated with negative psychological health outcomes^36^. The three scales yield acceptable internal consistency in the current sample (Reappraisal: α = 0.746; Suppression: α = 0.758; Externalizing Strategies: α = 0.724).

### Experimental emotion regulation paradigm

The emotion regulation paradigm resembles paradigms used previously in ER research^62,107,108^. Film stimuli used for the current paradigm consisted of two sadness inducing, two threatening, two happy, and one neutral film clips. The films had been previously validated to elicit the respective emotions^109,110^. The ER paradigm consisted of a passive viewing part and an ER part. Prior to the beginning of the passive viewing part, participants were instructed to keep sitting quietly, to breathe regularly, to passively view the film clips and to concentrate on the emotion they elicit. Then, a black screen was presented for 3 min to accommodate the participants to the situation and to assess a physiological baseline. After that, a neutral and one randomly chosen sad, one fearful and one happy clip were presented in random order. After each of the four film clips, a one-minute interstimulus interval (ISI) was presented. During this ISI participants were asked to indicate how intensely the film clips elicit a specific emotion by rating the intensity of the six basic emotions^111^ on visual analogue scales (range 0 = I did not feel the emotion at all, 100 = I extremely felt the emotion). In addition, patients rated how they felt while watching the film clips for valence (visual analogue scales, 0 = very negative, 100 = very positive) and arousal (visual analogue scales, 0 = not at all arousing, 100 = very arousing). This rating procedure had a duration of approximately 30 s. Then a black screen was presented for the rest of the one-minute ISI. After the passive viewing part had ended, participants were told that they will now see another sad and another threatening film clip. Contrary to the passive viewing part, they were now instructed to down-regulate the emotions elicited by the film. Reappraisal instructions were similar to those used in previous research^107^. In detail, participants were told the following: “We will now be showing you two further film clips. It is important to us that you watch the film clips carefully. This time, please try to adopt a detached and unemotional attitude as you watch the film. In other words, as you watch the film clips, try to think about what you are seeing objectively, in terms of the technical aspects of the events you observe. Watch the film clips carefully, but please try to think about what you are seeing in such a way that you don’t feel anything at all.” After the instruction, the two film clips were presented in random order, with an ISI of 1 min. Again, participants were asked to give a rating of the six basic emotions as well as for valence and arousal.

### Physiological recordings

M. corrugator supercillii EMG was recorded (sampling rate 1000 Hz, digitization 16 bit, Biopac MP100 amilifier system) from standard electrode sites^112^ using Ag/AgCl electrodes (inner diameter 5 mm). A ground electrode was attached to the participtant’s forehead. Online, data were notch filtered (50Hz). Offline, EMG data were bandpass filtered (28–500 Hz, 24 dB/oct, see^113^, and smoothed (moving average, width 20 ms). Offline, EMG data were averaged for the duration of the film clips and baseline corrected (i.e. with respect to the three-minute baseline-recording prior to the beginning of the paradigm).

### Treatments

The patients received Cognitive Behavior Therapy as usually carried out in our outpatient center (treatment-as-usual). Treatments typically include techniques such as behavior analyses, contingency management, cognitive restructuring, role play, relaxation trainings, etc. In terms of anxiety disorders, treatments typically additionally include exposure. The treatments comprised of approximately 25 sessions (*M* = 25.5, *SD* = 4.1). Sessions took place weekly. Treatments were carried out by therapists as part of their postgraduate training. All therapists had a master’s degree in psychology and at least 1-year full-time postgraduate CBT training. They were additionally monitored by licensed CBT supervisors within regular supervision sessions (i.e., including discussions about the patient’s status and the ongoing treatment). However, despite general agreement with published manuals treatments within routine outpatient care are usually less standardized than in typical randomized controlled trials^90^. All treatments were paid for by the German health care insurance system. The general effectiveness of the CBT treatments at our center have been demonstrated previously^90^ and specifically for the current patients’ sample^114^.

### Data analyses

#### Proof of principle experimentally assessed ER

To assess, whether participants were able to successfully down-regulate their emotions, repeated measures ANOVAs were calculated including the within subject independent variable condition (i.e. passive viewing vs. emotion regulation) for the threatening and the sad films respectively. This was done for M. corrugator supercilii activity, as well as subjective ratings of valence, arousal, emotion intensity ratings of sadness (for the sadness inducing film clip) and anxiety (for the anxiety inducing film clip). We found significant effects of ER on emotion intensity ratings, emotion arousal ratings, as well as for M. corrugator supercilii EMG activity. We did not find any significant effects of ER on valence ratings for neither of the film clips, thus valence ratings were discarded from further analyses. For those dependent variables showing successful emotion regulation in the proof of principle analyses (i.e. emotion intensity ratings, emotion arousal ratings, as well as for M. corrugator supercilii EMG activity), we then obtained emotion regulation ability scores by subtracting emotional reactivity during the emotion regulation condition from emotional reactivity during the passive viewing condition (i.e. passive viewing—emotion regulation). Greater values thus represent better emotion regulation ability. We then combined these variables to an emotion regulation ability score separately for the threatening and the sad ER conditions. Therefore, emotion intensity ratings, emotion arousal ratings and M. corrugator supercilii EMG activity were z-transformed and averaged. These ER ability scores represent the participants mean actual ability to regulate sad or threatening emotions (ER_sad_, ER_threat_) and were used for further analyses.

#### Cross sectional transdiagnostic association of ER with psychopathology

To assess the association between symptom severity and ER indices in the transdiagnostic sample, we first calculated Pearson correlations between the Reappraisal, Suppression and Externalizing Behavior subscale of the NARQ and the two ER ability scores (ER_sad_, ER_threat_) and the DASS depression, anxiety, and stress subscales of the DASS. Then, a series of linear regression analyses were run with the *Depression*, *Anxiety*, and *Stress* subscales as dependent variables, respectively. For each of the three DASS subscales, the two remaining subscales were entered as independent variables at level 1 (e.g. anxiety and stress were entered as independent variables when depression was the dependent variable). Then at level 2, ER ability scores (ER_sad_, ER_threat_) and the *Reappraisal*, *Suppression*, and *Externalizing Behavior* subscales of the NARQ were entered as independent variables. With this approach, we were able to assess the unique variance each of the three emotion disorders symptom clusters (i.e. depression, anxiety, stress) has in common with the five ER indices assessed in the current study.

#### Longitudinal prediction of treatment outcome with ER

Prediction of treatment outcome was done using linear regression analyses. Symptom change during treatment was operationalized using the DASS subscales *Depression*, *Anxiety* and *Stress*, as filled in by the patients prior to the beginning and after finishing their CBT treatment (pre-post treatment). Hierarchical linear regression analyses were run for each of the three DASS subscales respectively. Post-treatment levels of *depression*, *anxiety* and *stress* were used as dependent variables. For each of the three analyses pre-treatment scores of the respective DASS subscale (i.e. Pre-treatment *depression*, *anxiety* or *stress*) was entered as independent variable into the regression equation at level one. Then, the two ER ability scores (ER_sad_, ER_threat_) and questionnaire assessed ER strategy use (Reappraisal, Suppression and Externalizing Behavior subscales of the NARQ) were entered as independent variables at level two. Thus, pre-treatment symptoms are controlled for during calculation of regression weights for the prediction of post-treatment symptom severity. This approach strictly follows suggestions as formulated previously^115^ for studies predicting treatment outcome. With these analyses, it is possible to gather a precise estimate of the amount of variance explained by pre-treatment symptom level (the predictor forced to enter the regression first), as well as the variance explained by the predictor variables of interest (the variables forced to enter the regression after the pre-treatment score, in this case our ER indices).

#### General analyses remarks

Due to technical reasons, M. corrugator supercilii data of n = 28, DASS data of n = 3, NARQ data of n = 33, and rating data of n = 8 participants is missing. Additionally, several patients prematurely terminated their treatments or were unavailable for the post treatment questionnaire assessment (i.e. n = 58). Therefore, post-treatment DASS data was unavailable for n = 28 depressive and n = 30 anxiety patients. However, patients terminating prematurely did not differ from the remaining patients in any of the current ER measures (Er_sad_*p* = 0.358, ER_threat_, p = 0.588, NARQ_reappraisal_*p* = 0.600, NARQ_suppression_*p* = 0.086, NARQ_externalizing_*p* = 0.210), or in terms of diagnose, age or gender^114^.

All current statistics were calculated using IBM SPSS (Version 29). An alpha level of 0.05 was used for all statistical tests. For all ANOVAs eta squared effect sizes were calculated. Wherever possible, confidence intervals were calculated. For all regression analyses variance inflation factors were calculated to control for collinearity across predictor variables.

## Supplementary Information

Supplementary Table S1.

## Data Availability

The data that support the findings of this study are available from the first author upon reasonable request.
