# Supplementary material for: Differential effects of trait-like emotion regulation use and situational emotion regulation ability across the affective and anxiety disorders spectrum: a transdiagnostic examination
Source: Sci Rep. 2024 Nov 4;14:26642. doi: 10.1038/s41598-024-76425-7 (PMC11535244; doi:10.1038/s41598-024-76425-7)
Supplement: Supplementary file 1 — Supplementary Table S1. [file 41598_2024_76425_MOESM1_ESM.docx]

Table S1. Descriptive statistics of demographic variables and symptomatology for the three diagnostic groups

|  | ***DEP*** | ***ANX*** | ***HC*** | ***Group Comparisons*** |
| --- | --- | --- | --- | --- |
| **Age** *M (SD)* | 39.54 (14.68) | 34.14 (11.91) | 33.19 (11.29) | *F*(2,249) = 6.04, *p* = .003^1,3^ |
| **Sex** *(m/f)* | 42/61 | 39/54 | 14/64 | *χ*^2^ (2) = 6.40, *p* .041^1,2^ |
| **Psychotropic medication** *(yes/no)* | 56/47 | 33/60 | 0/60 | *χ*^2^ (2) = 49.45, *p* <.001^1,2,3^ |
| **DASS _General Distress_** *M (SD)* | 28.33 (12.21) | 23.67 (10.96) | 9.32 (7.64) | *F*(2,250) = 52.17, *p* < .001^1,2,3^ |
| **DASS _Depression_** *M (SD)* | 11.34 (5.99) | 7.20 (5.07) | 2.32 (2.49) | *F*(2,250) = 58.73, *p* < .001^1,2,3^ |
| **DASS _Anxiety_** *M (SD)* | 5.62 (4.30) | 6.46 (4.47) | 1.66 (1.89) | *F*(2,250) = 27.34, *p* < .001^1,2^ |
| **DASS _Stress_** *M (SD)* | 11.47 (4.96) | 10.59 (5.03) | 5.34 (4.16) | *F*(2,250) = 31.38, *p* < .001^1,2^ |

Note: HC=healthy Controls, DEP=Depressed Patients, ANX=Anxiety Patients, Significant group comparisons (i.e. p<.05): 1=HC vs. DEP, 2=HC vs. ANX, 3=DEP vs. ANX; (number in brackets indicate significance at trend level, i.e. p<.10), DASS = Depression Anxiety and Stress Scale

**Conceptual fit of the Depression Anxiety and Stress Scale with transdiagnostic models of depression and anxiety.**

We chose to use the Depression, Anxiety and Stress Scale (DASS) [1] because of its’ conceptual proximity to the tenets of the tripartite model of anxiety and depression [2]. As an early transdiagnostic model, the tripartite model was empirically derived and developed to explain comorbidity between depression and anxiety disorders on a symptom level. As such, the conceptual foundations of the tripartite model are closely related to more recent transdiagnostic approaches to anxiety and affective disorder psychopathology, in specific to the internalizing spectrum described in the Hierarchical Taxonomy of Psychopathology (HiTOP) [3]. Importantly, the DASS has been developed to assess core symptoms of anxiety and depression while providing maximum discrimination between the subscales [1,4]. Although the DASS was developed independently from Clark and Watson's tripartite model of anxiety and depression, the three psychometrically distinct DASS factors could be regarded as a proxy for the three components of the tripartite model: DASS-Depression: assessing low positive affect, loss of self-esteem and incentive, hopelessness (absence of positive affect); DASS-Anxiety: assessing autonomic arousal and fearfulness (physiological hyperarousal); and DASS-Stress: assessing tension, irritability, and a low threshold for becoming upset / frustrated (negative affect) [1]. Thus, using the DASS maximizes discrimination of symptom clusters as well as conformity with empirically derived theoretical and transidagnostic models of depression and anxiety. In sum, the DASS is well suited to assess the core symptoms within a transdiagnostic approach across the affective and anxiety disorders spectrum.

**References**

1. Lovibond, P. F. & Lovibond, S. H. (1995). The structure of negative emotional states: Comparison of the Depression Anxiety Stress Scales (DASS) with the Beck Depression and Anxiety Inventories. *Behaviour Research and Therapy* 33, 335–343. Doi: [10.1016/0005-7967(94)00075-U](https://doi.org/10.1016/0005-7967(94)00075-U).
2. Clark, L. A. & Watson, D. (1991). Tripartite model of anxiety and depression: psychometric evidence and taxonomic implications. *Journal of Abnormal Psychology* 100, 316–336.
3. Kotov, R., Krueger, R. F., Watson, D., Achenbach, T. M., Althoff, R. R., Bagby, R. M., ... & Zimmerman, M. (2017). The Hierarchical Taxonomy of Psychopathology (HiTOP): A dimensional alternative to traditional nosologies. *Journal of Abnormal Psychology*, *126*(4), 454-477. Doi: [10.1037/abn0000258](https://psycnet.apa.org/doi/10.1037/abn0000258).
4. Brown, T. A., Chorpita, B. F., Korotitsch, W. & Barlow, D. H. (1997). Psychometric properties of the Depression Anxiety Stress Scales (DASS) in clinical samples. *Behaviour research and therapy*, *35*(1), 79-89. Doi: *0005-7967* 35.
